# Neurological Benefits, Clinical Challenges, and Neuropathologic Promise of Medical Marijuana: A Systematic Review of Cannabinoid Effects in Multiple Sclerosis and Experimental Models of Demyelination

**DOI:** 10.3390/biomedicines10030539

**Published:** 2022-02-24

**Authors:** Victor Longoria, Hannah Parcel, Bameelia Toma, Annu Minhas, Rana Zeine

**Affiliations:** 1 Basic Medical Sciences, St. Vincent Campus, Saint James School of Medicine, 1480 Renaissance Drive, Park Ridge, IL 60068, USA; vlongoria@mail.sjsm.org (V.L.); hparcel@mail.sjsm.org (H.P.); btoma@mail.sjsm.org (B.T.); aminhas@mail.sjsm.org (A.M.); 2 School of Natural Sciences, Kean University, 1000 Morris Ave., Union, NJ 07083, USA

**Keywords:** medical marijuana, cannabinoids, cannabidiol (CBD), delta-9-tetrahydrocannabinol (Δ^9^-THC), multiple sclerosis (MS), experimental autoimmune encephalomyelitis (EAE), Theiler’s murine encephalomyelitis virus-induced demyelinating disease (TMEV-IDD), neuroinflammation, neuroprotection, remyelination

## Abstract

Despite current therapeutic strategies for immunomodulation and relief of symptoms in multiple sclerosis (MS), remyelination falls short due to dynamic neuropathologic deterioration and relapses, leading to accrual of disability and associated patient dissatisfaction. The potential of cannabinoids includes add-on immunosuppressive, analgesic, neuroprotective, and remyelinative effects. This study evaluates the efficacy of medical marijuana in MS and its experimental animal models. A systematic review was conducted by a literature search through PubMed, ProQuest, and EBSCO electronic databases for studies reported since 2007 on the use of cannabidiol (CBD) and delta-9-tetrahydrocannabinol (THC) in MS and in experimental autoimmune encephalomyelitis (EAE), Theiler’s murine encephalomyelitis virus-induced demyelinating disease (TMEV-IDD), and toxin-induced demyelination models. Study selection and data extraction were performed by 3 reviewers, and 28 studies were selected for inclusion. The certainty of evidence was appraised using the Cochrane GRADE approach. In clinical studies, there was low- and moderate-quality evidence that treatment with ~1:1 CBD/THC mixtures as a nabiximols (Sativex^®^) oromucosal spray reduced numerical rating scale (NRS) scores for spasticity, pain, and sleep disturbance, diminished bladder overactivity, and decreased proinflammatory cytokine and transcription factor expression levels. Preclinical studies demonstrated decreases in disease severity, hindlimb stiffness, motor function, neuroinflammation, and demyelination. Other experimental systems showed the capacity of cannabinoids to promote remyelination in vitro and by electron microscopy. Modest short-term benefits were realized in MS responders to adjunctive therapy with CBD/THC mixtures. Future studies are recommended to investigate the cellular and molecular mechanisms of cannabinoid effects on MS lesions and to evaluate whether medical marijuana can accelerate remyelination and retard the accrual of disability over the long term.

## 1. Introduction

Patient dissatisfaction with multiple sclerosis (MS) treatments continues to motivate the search for adjunctive remedies in the hope of preventing breakthrough relapses and worsening of disability. Neurologic dysfunction and accrual of disability in multiple sclerosis patients are attributed to the accumulation of white matter and cortical demyelinative lesions over time, with subsequent neuronal loss, associated with increasing inflammation in the brain, spinal cord, or leptomeninges [1,2,3]. Intervals of remission between the relapses [4] allow for partial remyelination and periodic halting of impairment worsening.

MS patients manifest a range of symptoms [5] as a result of motor, sensory, autonomic, and psycho-behavioral dysfunction including fatigue (75–90%) and mobility impairment (90%) [6], gait difficulties [7], paresthesia [8], vision problems [9,10], speech impairments [11,12], dizziness and vertigo [13], urinary bladder dysfunction [14,15], neurogenic bowel dysfunction [16], sexual dysfunction [17], chronic neuropathic pain (85%) [18], cognitive deficits in information processing, speed, episodic memory, complex attention and executive function [19,20,21], physical disability [22], anxiety and depression [23], and sleep disturbances that correlate with fatigue and depression [24,25,26].

### 1.1. Clinical Subtypes of MS

Individuals who present with a clinically isolated syndrome (CIS) who also have MRI findings consistent with active inflammation in another region of the central nervous system suggestive of an earlier episode are considered at high risk for developing relapsing-remitting MS (RRMS) and may be treated with moderate-efficacy disease-modifying therapies, such as interferon-β (Avonex, Rebif, Extavia) and the mixture of synthetic polypeptides glatiramer acetate (Copaxone, Copolymer 1), to delay the development of clinically definite MS [27].

A relapsing-remitting disease course develops initially in ~85% of patients diagnosed with MS as they experience varying degrees of functional impairment that may or may not worsen following each relapse [28,29,30]. Eventually, a proportion of these patients transition to secondary progressive MS (SPMS) as they continue to accrue disability but have fewer or no remissions. The remaining ~15% of patients never experience any relapses or remissions as they exhibit continuous worsening of neurologic deficits starting from the initial onset of their symptoms and are therefore given the diagnosis of primary progressive MS (PPMS) [28,29].

In the presence of early-onset substance abuse, alcohol dependence, and physical and/or mental comorbidities, the diagnosis of MS may be delayed by several years [31]. 

### 1.2. Clinicopathologic Correlations in MS

Pathologic examination of central nervous system tissues from autopsy patients with primary and secondary progressive MS shows that they carry a higher demyelinative lesion load, which correlates with greater disease severity and shorter time to disability as assessed on the Expanded Disability Status Scale (EDSS), as compared to relapsing MS [32]. Concurrently, the proportion of lesions showing evidence of remyelination is lowest in primary progressive MS and highest in relapsing-remitting MS [32]. Thus, neuroinflammation, loss of myelin, and neurodegeneration all contribute to the accumulation of permanent disability in MS patients. 

The anti-inflammatory effects of disease-modifying therapies, including immunomodulatory monoclonal antibodies, help to reduce relapse frequency and delay disease progression in patients with MS, while a variety of other symptom-altering drugs, including the inhibitory neurotransmitter γ-aminobutyric acid (GABA)-B receptor agonist baclofen [33] and the α2-adrenergic receptor agonist tizanidine, help to improve neuropathic pain, spasms, spasticity, urinary bladder dysfunction, bowel dysfunction, sexual dysfunction, gait difficulties, dizziness, vertigo, fatigue, and depression [28,34]. Regardless of the number of years of MS duration, three types of demyelinative lesions, namely, “active”, “inactive”, and “mixed active/inactive” (or chronic active), are detected in varying proportions and distinguished based on the level and pattern of innate immune activity observed [1,32]. The proportions of “inactive” lesions, those that are entirely demyelinated and devoid of human leukocyte antigen (HLA)-expressing macrophages/microglia, may be highest in populations with decades-long disease durations [32]. Nevertheless, both “active” lesions, in which HLA+ macrophages/microglia are present throughout the demyelinated areas, and “mixed active/inactive” lesions, in which HLA+ macrophages/microglia are limited to the edges bordering hypocellular centrally gliotic demyelinated areas, may still account for about one third of all lesions found at the time of death after long-standing illness [32]. The type of lesion which most strongly correlates with a greater disease severity and more rapid progression was found to be the “mixed active/inactive” lesions, which also inversely correlate with the extent of remyelination [32]. This recalcitrant neuropathologic picture is reflected in three descriptors that neurologists use to document clinical disease status in MS patients: (a) clinical or MRI “activity” which may be detected over a specified period of time, (b) worsening disability that may be documented following a relapse, and (c) ongoing disease progression that may be interrupted by periods of disease stability [30]. 

### 1.3. Prevalence and Risk Factors of MS

The global prevalence of MS was 35.9 per 100,000 in 2020, with an estimated 2.8 million people living with MS worldwide [35]. Females are twice as likely to have MS as males globally; however, in some countries, the ratio of women to men reaches 4:1 [35,36,37].

MS exhibits heterogeneity with respect to clinical, genetic, radiographic, and pathologic features [38]. Triggers for MS development and relapses involve interactions between genetic, lifestyle, and environmental factors [39]. Genome-wide association studies (GWAS) have identified over 230 genetic risk loci for MS, revealing a ~5-fold increase in the risk of MS when the presence of the class II variant HLA-DRB1*15:01 is combined with an absence of the class I variant HLA-A*02 [40]. Other risk factors for MS include smoking, alcohol consumption, obesity, low dietary intake of vitamin D, lower sun exposure, latitude farther away from equator, and certain chronic viral infections [39]. Longitudinal analysis using data from US military recruits over a period of 20 years has revealed that Epstein–Barr virus (EBV) increased the risk of subsequent MS by 32-fold [41]. MS patients have significantly higher serum anti-EBV nuclear antigen-1 (EBNA-1) titers as compared to healthy controls, and the higher EBV responses correlate with more extensive lesion and gray matter tissue destruction as measured by magnetization transfer imaging in RRMS patients [42]. Molecular mimicry may explain the association between EBV titers and more severe neuropathology since ~20–25% of MS patients have anti-EBNA1 antibodies that cross-react with the CNS protein glial cell adhesion molecule (GlialCAM) [43].

In humanized non-obese diabetic-severe combined immunodeficiency (NOD-scid) IL2 receptor γ-chain-deficient (huNSG) mice, EBV infection was found to synergize with HLA-DR15 by priming cross-reactive CD4^+^ T-cell clones which control the viral infection less efficiently [44]. It is hypothesized that EBV might activate ancestral human endogenous retroviruses (HERVs) in the human genome [45,46,47]. Antibodies to other viruses have been associated with increased MS conversion and relapse including human herpes virus 6 (HHV-6) [48]. It is believed that the induction of demyelination in the brain and spinal cord in MS may be initiated by excess innate and myelin-specific autoimmune activation mechanisms that are sensitive to chronic viral infections and gut microbiome status [49] and perpetuated by the loss of oligodendrocytes and their progenitors through either perforin-mediated lysis [50,51,52] or apoptotic cell death [53]. 

### 1.4. Experimental Animal Models of MS

Animal models of MS include experimental autoimmune (allergic) encephalomyelitis (EAE), Theiler’s murine encephalomyelitis virus-induced demyelinating disease (TMEV-IDD), and toxin-induced demyelination models [54,55]. The clinical severity of EAE is scored as either (0) no overt signs, (1) either floppy tail or hindlimb weakness, (2) both limp tail and hindlimb paresis, (3) hindlimb paralysis, (4) forelimb and hindlimb paralysis, and (5) moribund.

Environmental triggers and disease onset are best studied in spontaneous EAE transgenic models in which gene expression has been linked to risk genes of MS [56].

Neuroinflammation and immunomodulation are best studied using EAE models [57]. Chronic EAE is actively induced by immunization of C57BL/6 mice with myelin oligodendrocyte peptide (MOG_35–__55_) [58], with options for utilizing transgenic mice that provide specific immunogenetic backgrounds [55,59]. A remitting-relapsing form of EAE is actively induced by immunization of SJL/J mice with intact protein or peptides from either myelin basic protein (MBP) [60,61,62,63] or proteolipid protein (PLP_139–151_) [64]. Monophasic EAE can be induced by immunization of Lewis rats with MBP_68–86_, and chronic disease can be induced in common marmoset and rhesus macaque non-human primates with MOG_34–56_ [55,65,66]. EAE can also be adoptively transferred by injection of myelin-reactive CD4^+^ T cells into naïve mice. 

Demyelination, neurodegeneration, and remyelination are best studied using TMEV-IDD, in which Theiler’s virus infects oligodendrocytes, microglia, and astrocytes, causing a monophasic fulminant demyelinating disease with motor function deficits [67,68], Cuprizone-induced demyelinative disease, in which mice are fed with a pelleted diet containing 0.2% Cpz (Bis(cyclohexanone)oxaldihydrazone) [69,70], or in vitro lysolecithin-induced demyelination [71] models.

### 1.5. Therapeutic Challenges and Biomarker Research in MS

Among first-line pharmacotherapies in MS is glatiramer acetate; however, the response rate is not greater than 55% due to variability in genetic polymorphisms [72]. Recent studies have demonstrated that long-term disability outcomes tend to be better in MS patients who are treated early with “high-efficacy” medicines including the anti-CD20 monoclonal antibodies rituximab (Rituxan) and ocrelizumab (Ocrevus) targeting B cells, the anti-α4β1 integrin monoclonal natalizumab (Tysabri), the type II topoisomerase inhibitor mitoxantrone (Novantrone), the sphingosine-1-phosphate receptor modulator fingolimod (Gilenya) [73], the monoclonal anti-CD52 alemtuzumab (Campath, Lemtrada) targeting mature lymphocytes, and the purine analogue cladribine (Mavenclad) that delivers “intensive” immune intervention, as compared to patients who are treated with “moderate-efficacy” medications for one or more years prior to “escalation” to the high-potency agents [74,75]. Adverse effects are more complex for the “high-efficacy” treatment strategies and include increased risks of serious infections and cancers associated with immunosuppression, autoimmune diseases associated with immunomodulation, cardiotoxicity, hepatotoxicity, and gastrointestinal and neuropsychiatric disorders [76,77]. 

A hallmark of MS is the presence of oligoclonal bands in the cerebrospinal fluid or an elevated immunoglobulin G (IgG) index found in up to 95% of patients [78]. One biomarker for long-term MS activity and disease progression has been shown to be low serum vitamin D [79]. Oxidative and antioxidative enzymes as well as redox degradation products have also been found to be possible biomarkers for MS, and correlations have been identified between clinical subtypes of MS and levels of superoxide dismutases (SOD), 3-NO-Tyr, and the lipid peroxidation marker malondialdehyde (MDA) [80]. Biomarker research goals include determining the usefulness of tNOx, S-nitrosothiol, SOD, myeloperoxidase (MPO), glutathione-disulfide reductase (GSR), catalase, protein carbonyls, advanced oxidation protein products (AOPPs), F2-isoP, MDA, and oxycholesterols as potential prognostic biomarkers for MS, determining the usefulness of SOD, nuclear factor erythroid 2-related factor 2 (Nrf2), protein carbonyls, 3-NO-Tyr, AOPPs, and MDA as possible predictive biomarkers for response to treatment, and investigating SOD as a therapeutic drug target in MS [63,80].

Another potential biomarker that may be useful in screening for MS is low serum and CSF tryptophan (TRP) levels because kynurenic acid (KYNA), a product of L-tryptophan metabolism through the kynurenine pathway in the CNS, is a competitive antagonist of NMDA, AMPA, and kainite excitatory glutamate receptors, with antioxidative, anti-inflammatory, and neuroprotective properties [81,82].

### 1.6. The Potential of Cannabinoids in MS

Sadly, MS disease progression still cannot be prevented. Of 5481 MS patients surveyed in 2014 by the North American Research Committee on Multiple Sclerosis (NARCOMS), 47% had considered using marijuana for their MS, 20% had spoken with their physician about using it, and 26% had used it for their MS [83]. Animal studies demonstrating the ability of cannabinoid treatment to reduce neuroinflammatory infiltration and improve hindlimb spasticity in EAE [84,85,86,87], and to confer neuroprotection [88], have prompted interest in investigating their potential benefits in human MS [89,90,91,92,93,94,95]. Early studies also demonstrated that cannabinoids could ameliorate clinical progression, downregulate proinflammatory T cells, and promote remyelination in TMEV-IDD [96,97]. The therapeutic potential of cannabis and cannabinoids relates to the multifunctional capacities of the endocannabinoid receptor system in neuroprotection, regeneration, and remyelination in addition to anti-inflammatory effects [98,99,100]. The hope of reproducing in human MS the pro-repair effects that have been observed in CNS tissues in a variety of in vitro and in vivo experimental systems offers the possibility of gaining neuropathologic benefits that extend beyond the relief provided by immunosuppression alone. 

Clinical studies suggest that combinations of the cannabinoids derived from the *Cannabis sativa* plant, cannabidiol (CBD) and Δ^9^-tetrahydrocannabinol (Δ^9^-THC), are comparably as effective for short-term symptomatic relief as conventional pharmacotherapeutic agents while causing less side effects [101]. An oromucosal spray formulation, Sativex^®^ (nabiximols), which contains Δ^9^-THC and CBD in a nearly 1:1 ratio, was licensed in the United Kingdom in 2010 as a prescription-only medicine for the treatment of spasticity in multiple sclerosis. 

### 1.7. Reviewing Human and Animal Studies

The first branch of this paper systematically reviews the findings of studies in the last 15 years of cannabinoid treatment effects on spasticity, pain, bladder overactivity, sleep disturbances, and quality of life in human MS. Cognitive deficits were not the primary outcomes measured in these studies, perhaps because the common adverse effects of cannabinoid treatment include dizziness and somnolence that could either mask any benefits to cognition or exacerbate any baseline cognitive deficits. Long-term disability was also not possible to assess because the studies were short term.

Because animal studies offer more detailed mechanistic information about the pathobiology of neuroinflammation and remyelination than is possible with human studies, the second branch of this paper reviews the effects of cannabinoid use in several experimental animal models of MS. Studies assessed measures of clinical disease severity, CNS inflammatory infiltration, microglial activation, neuroprotection, demyelination, and remyelination in actively induced and adoptively transferred EAE, Theiler’s murine encephalomyelitis virus-induced demyelinating disease (TMEV-IDD), and toxin-induced demyelination models. Studies on other experimental models of toxic, traumatic, or viral demyelinative brain injury were also discussed in the context of elucidating possible cellular and molecular mechanisms of cannabinoid-mediated CNS repair. 

## 2. Methods

A systematic review was conducted in accordance with the PRISMA 2020 Statement Guidelines [102]. A literature search was performed through PubMed, EBSCO Host, and ProQuest electronic databases. The text words “medical marijuana”, “cannabis”, “cannabinoids”, “cannabidiol”, “CBD”, “CBD-1 receptor”, “CBD-2 receptor”, “Δ^9^-Tetrahydrocannabinol”, “Δ^9^-THC”, “disease-modifying drugs”, and “immunomodulatory medications” with the use of the Boolean operator “AND” “multiple sclerosis”, “relapsing MS”, “progressive MS”, “MS lesions”, “experimental allergic encephalomyelitis”, “EAE”, “experimental autoimmune encephalomyelitis”, “Theiler’s murine encephalomyelitis virus”, “TMEV”, “cuprizone-induced demyelinative disease”, “lysolecithin-induced demyelination, ”demyelination”, “remyelination”, “spasticity”, “pain”, “sleep”, “urinary bladder”, “incontinence”, and “inflammation” were used to identify studies discussing the effectiveness of medical marijuana in patients with multiple sclerosis and its animal models. 

**Figure 1:**
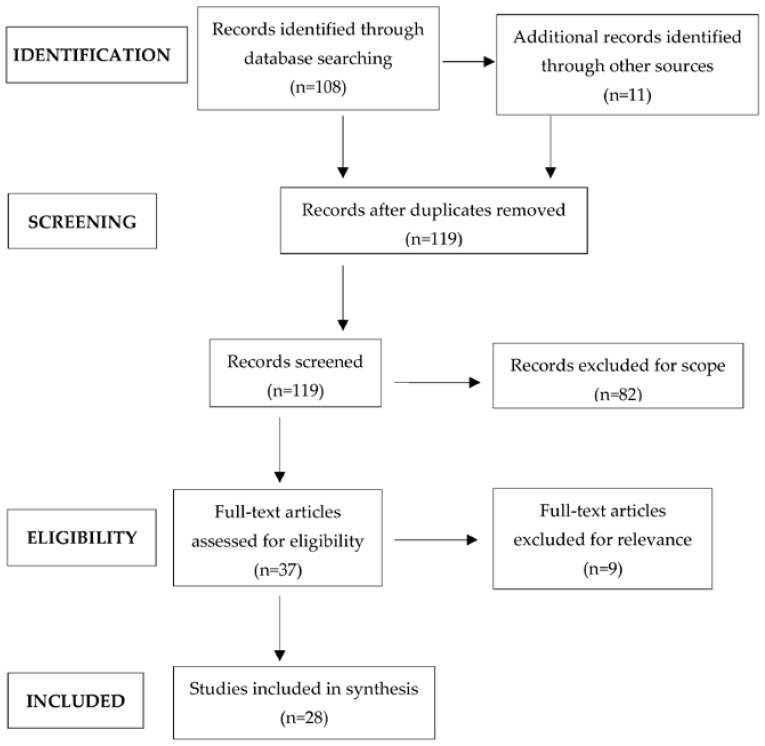
PRISMA flow chart for search strategy.

The search strategy is shown in the PRISMA flow chart (Figure 1). Inclusion criteria included the following: (1) must be a scholarly or peer-reviewed source, (2) article within the last 15 years, and (3) articles available in the English language. Exclusion criteria were the following: (1) publications potentially used for marketing purposes, (2) articles earlier than 2007, and (3) articles not available in English. 

Data extraction was performed by 3 reviewers, and 28 studies were selected for inclusion. The methodological quality of each included study was assessed based on the Cochrane Risk of Bias tool. Primary outcome measures included changes, if any, in muscle spasticity, pain/neuropathic pain, quality of sleep, neurogenic urinary bladder functions, and inflammation. Secondary outcomes included changes in disability status, adverse effects, and drug interactions. The articles were rated according to the following scheme: (1) properly conducted randomized controlled clinical trial (RCCT); (2) well-designed controlled trial without randomization, or prospective comparative cohort studies; (3) case–control studies, or retrospective cohort study; (4) cross-sectional study; and (5) case series, or observational (clinical or experimental) studies. The certainty of evidence for each outcome was assessed per human study using the Cochrane GRADE (Grading of Recommendation Assessment, Development and Evaluation) approach by evaluating eight factors: (a) Risk of Bias, (b) Inconsistency, (c) Indirectness, (d) Imprecision, (e) Publication Bias, (f) Dose–Response Relationship, (g) Size of Effect, and (h) Confounding [103,104]. The quality of evidence was rated as either high, moderate, low, or very low by GRADE categories. 

## 3. Results

Altogether, 119 articles were identified and screened. After exclusion of 82 records, 37 studies were assessed for eligibility, of which 28 were included in the synthesis, i.e., 14 human and 14 animal studies. 

Outcome data extracted from studies conducted on populations with multiple sclerosis were organized by the neurologic symptoms of spasticity (Table 1), pain (Table 2), neurogenic bladder function (Table 3), and sleep disturbance (Table 4). Results extracted from animal studies of experimental autoimmune (allergic) encephalomyelitis (EAE), TMEV-induced chronic demyelinating disease, and toxin-induced demyelination included measures of inflammation, neuroinflammatory infiltrates, clinical scores, spasticity evaluated by hindlimb stiffness, motor function, and measures of remyelination and axonal regeneration (Table 5 and Table 6). 

In clinical studies, changes in spasticity were assessed in human MS using either the Modified Ashworth Scale (MAS) for muscle tone, on which a score of 4 indicates rigidity of affected parts in flexion or extension, or the numerical rating scale (NRS), on which scores of 7–10 indicate severe spasticity. Spasticity in EAE was evaluated by the “stiffness” of limbs as assessed by the resistance force against hindlimb flexion. Motor function in TMEV-IDD and Cpz-induced demyelination was evaluated by rotarod and by vertical and horizontal activity measurements.

Changes in self-reported pain intensity were assessed using either an 11-point numerical rating scale (NRS) for pain, on which a score of 10 represents the most severe pain, a verbal rating scale (VRS), or a visual analog scale (VAS), on which degrees of pain intensity are described using adjectives such as “none”, “mild”, “moderate”, or “severe”. 

Effects on lower urinary tract dysfunctions related to neurogenic overactive bladder were evaluated using the number of voids per day, the number of nocturia episodes, overall bladder condition (OBC), overactive bladder symptom score (OABSS), post-void residual volume (PVR), patient’s global impression of change (PGIC), International Prostatic Symptoms Score (IPSS), or urinary incontinence quality of life (I-QOL).

Effects on sleep disturbances were assessed using either the NRS for sleep, on which 0 represents the best and 10 indicates the worst quality of sleep in a 24 h period, or the Pittsburg Sleep Quality Index, which is a one-month self-rated questionnaire. Self-reported, survey-based assessments utilized NIA-NIH Patient Reported Outcomes Measurement Information System (PROMIS) forms for pain intensity, pain interference, depression, anxiety, fatigue, sleep disturbance, and cognitive abilities.

Changes in disability status were assessed using EDSS or parameter-specific quality of life (QOL) evaluation instruments.

In preclinical studies, changes in lymphocytic inflammatory infiltrates, cytokines, chemokines, myelination, and axonal regeneration in brain and spinal cord specimens collected from experimental animals were quantified by histologic, immunohistochemical, immunofluorescence, flow cytometry, Western blot, ELISA, PCR, or sequence analysis.

## 4. Discussion

### 4.1. Certainty of Evidence for Medical Marijuana Effects on MS Symptom Outcomes

This systematic review of the literature evaluated the evidence for benefits of medical marijuana treatment in multiple sclerosis, and examined the effects of cannabinoids in autoimmune, viral, and toxic models of demyelination, over the past 15 years. 

A total of 14 animal studies were reviewed, 9 on EAE (Table 5), 3 on TMEV-IDD, and 2 on toxin-induced demyelination (Table 6). The experimental results combined adequately demonstrate that cannabinoid treatments are effective in diminishing clinical disease severity, alleviating hindlimb stiffness, facilitating recovery, improving motor function, strengthening anti-inflammatory responses, recruiting immunosuppressive cell populations, inhibiting leukocyte adhesion, enhancing lymphocyte apoptosis, lessening the degree of demyelination, providing neuroprotection, reducing axonal loss, and promoting remyelination in the CNS. One study on toxin-induced demyelination [127] showed a narrow therapeutic window for cannabinoid benefits reminiscent of that seen in the human studies. While internal validity was very good in the preclinical studies because experiments were well designed and well controlled, the external validity of animal studies is less certain due to differences in the cannabinoid systems between species that may affect safety, dose responses, tolerability, and homeostasis. For example, in several of the studies, animals responded to treatments with single agents, either CBD alone or Δ^9^-THC alone, which have been previously shown to have suboptimal effects and less tolerability in humans [131,132,133,134,135]. 

A total of 14 human studies were reviewed. Risk of bias assessment was performed for each of the human studies using the Cochrane criteria appropriate for appraising randomized human clinical trials and cohort studies. The quality of evidence was assessed using the Grading of Recommendations Assessment, Development and Evaluation (GRADE) approach [103,104], as presented per study in Table 7 for the certainty of evidence, and per outcome in Table 8 for the summary of findings.

Mean maximal change in spasticity NRS from baseline was −2.8 pts (min. −0.04, max. −7.4) between 12 wks and 12 mo.

Spasticity outcomes were reported in nine studies: three randomized, double-blind, placebo-controlled clinical trials, and six cohort/observational studies. The quality of evidence GRADE was moderate in six and low in three of the studies (Table 7 and Table 8). The total number of subjects for spasticity studies was 1582. The mean maximal change in NRS spasticity scores was 2.8 (range 0.04 to 7.4) lower than baseline. Assessments were repeated more than once within a period of 4 months, and two of the studies extended up to 12 months. In the three clinical trials, the mean difference in NRS spasticity scores was 0.62 (range 0.5 to 0.83) points lower in the treatment groups as compared to controls, as a placebo effect was noted in two of the trials. The dose delivered by an oromucosal spray of a THC/CBD 1:1 mixture was comparable in all of the studies, remaining within a narrow therapeutic window. Participants were allowed to up-titrate the number of puffs administered per day to reach a dose that was optimal for them but that did not exceed a maximum limit. 

Pain outcomes were reported in five studies: one randomized, double-blind, placebo-controlled clinical trial, and four clinical observational studies. The quality of evidence GRADE was moderate in two, low in two, and very low in one of the studies (Table 7 and Table 8). The total number of participants for pain studies was 573. By 4 weeks of treatment, the mean pain NRS score was lower by 3.42 points from baseline. In one cohort study, the benefit in pain reduction remained stable at 6 months. 

Lower urinary tract function outcomes were reported in three studies: one randomized, double-blind, placebo-controlled clinical trial, and two clinical observational studies. The quality of evidence GRADE was moderate in two and low in one of the studies (Table 7 and Table 8). The total number of subjects for urinary bladder dysfunction studies was 235. Although improvements in urinary incontinence had previously been reported with cannabis extract and THC [136], no significant improvement was found in the studies using the Sativex oromucosal spray. Modest reductions in bladder overactivity, however, were reported in measurements of the numbers of voids per day, urgency episodes per day, nocturia episodes per day, OBS NRS at 8 weeks, and bladder-related quality of life NRS scores, while neurophysiological studies demonstrated modest improvements in bladder dysfunction by the OABSS, PVR (50 mL) at 4 weeks, and IPSS at 6 months. Placebo effects were noted as well.

Sleep quality outcomes were reported in three studies: one randomized, double-blind, placebo-controlled clinical trial, and two clinical observational studies. The quality of evidence GRADE was moderate in one, low in one, and very low in one of the studies. The total number of participants for sleep studies was 816. Improvements were reported in quality of life, and to variable extents in quality of sleep. 

In all cannabinoid treatment groups, there were responders and non-responders. Some of the non-responders opted to withdraw from the studies for lack of benefit. Some of the responders opted to withdraw from the studies due to difficulties tolerating the adverse effects. For these reasons, attrition bias underpowered the studies, and intention-to-treat bias occasionally necessitated careful handling of data analyses. Additionally, knowledge of the allocated intervention by participants introduced risks of performance bias in all but one study. Although there was consistency in the direction of effects, placebo effects that were appropriately taken into consideration in the clinical trials were not available from cohort studies because of a lack of control cohorts. Consequently, the magnitudes of the clinical effects attributable to cannabinoid treatment in the cohort studies were most likely overestimates, and future studies without control cohorts should be strongly discouraged. A possible confounding factor in all of the studies was the concomitant use of one or more MS pharmacotherapeutic or other medications. 

The growing body of moderate-quality evidence for the safety and efficacy of cannabinoid treatment using 1:1 THC/CBD mixtures has led to its approval in some countries for the management of spasticity, pain, and bladder dysfunction in MS [137,138]. Our assessments agree with others, finding that the magnitudes of effects on short-term neurological outcomes in MS patients are either small, limited, or moderate, and that the benefits are more easily detected by subjective rather than objective measures [134,139,140,141]. It is noted that Sativex^®^ contains small amounts (<10%) of terpenes and flavonoids of the Cannabis plant that might influence the actions of the THC and CBD; however, studies are ongoing as new formulations become available and longer courses of treatment become sustainable. 

To increase confidence in the efficacy of medical marijuana as an add-on therapy in MS, higher-quality, multi-year, randomized, double-blind, placebo-controlled clinical trials are warranted to assess long-term tolerability, drug–drug interactions due to the known potential for the induction of cytochrome P450, benefits for preventing breakthrough relapses, ability to reverse deficits or retard the accrual of disability, and the impact on measures of quality of life. Radiological studies would be extremely valuable as follow-up measures since precise associations have been described between different types of CNS pathology and MRI findings in multiple sclerosis [142].

Cannabis-based medicine has long been known to be useful in pain management including central pain in multiple sclerosis and post-operative pain [143,144]. Promising benefits of cannabis use and medical marijuana have also been observed for relief of neurologic symptoms in patients with movement disorders, including Parkinson disease and Huntington disease [145]. Furthermore, oral CBD has been used for the treatment of drug-resistant seizures in children with tuberous sclerosis (TSC) [146], and Epidiolex^®^ (pure CBD) has been approved for the treatment of intractable epilepsy in patients with developmental epileptic encephalopathies [147] including Dravet syndrome and Lennox–Gastaut syndrome (LGS). Multiple mechanisms are implicated in the ability of CBD to modulate seizures that include antagonism of CB1, CB2, GPR18, GPR55, and voltage-gated sodium channel (VGSC) receptors; agonism of GABA_A_ receptors; activation and desensitization of TRPV1/2 receptors; and allosteric modulation of opioid receptor types μ and δ, leading to inhibition of glutaminergic N-methyl-D-aspartate (NMDA) receptors [148]. 

Cannabis-based extracts and CBD have been effective clinically in the management of sleep disturbances and anxiety in psychiatric patients as well as in MS [149,150]. A discontinuation study on MS patients has demonstrated that those who respond to cannabinoids could be identified within the first 6 weeks after starting treatment [151].

### 4.2. Elucidating the Molecular Mechanisms of Medical Marijuana That May Be Therapeutic in MS

To explore the cellular and molecular mechanisms through which cannabinoid-induced effects might favorably impact the prognosis in MS, additional experimental observational studies are required to investigate the exact types of receptors and signaling pathways that could reverse injury to oligodendrocytes and neurons and promote CNS tissue repair, regeneration, and remyelination [152,153].

Type 1 cannabinoid receptors (CB1) [154] and type 2 cannabinoid receptors (CB) [155] are eukaryote-specific, class A, G protein-coupled membrane receptors (GPCRs) that are widely distributed and highly expressed throughout mammalian tissues. Cannabinoid (CB) receptors are activated by the endogenous cannabinoids anandamide (AEA) [156] and 2-arachidonoylglycerol (2-AG) [157,158] to modulate neuronal excitability, synaptic neurotransmitter release throughout the CNS, and inflammatory processes [159,160,161]. 

CB1 receptors are found mainly on neurons in the neocortex, hippocampus, amygdala, basal ganglia outflow tracts, cerebellum, spinal cord, and peripheral nervous system (PNS) and are also expressed on astrocytes [162], oligodendrocytes, microglia [163], dendritic cells, the GI tract, liver, heart, adipose tissue, bone, skin, eyes, skeletal muscle, and reproductive system [159,160,161,164,165,166,167]. In the human brain, cannabinoid receptors are localized in areas of higher cognitive functions (forebrain), control of movement (forebrain, midbrain, and hindbrain), and control of motor and sensory functions of the autonomic nervous system (hindbrain) [168]. Activation of CB1 receptors inhibits GABA and glutamate release and increases the activity of potassium and calcium ion channels, thus affecting mood (euphoria), appetite, and nociception (analgesia), and mediating neuronal survival, axonal preservation, and the decrease in astrocyte extracellular matrix production. Δ^9^-THC binds CB1 receptors, acting as a partial agonist that downregulates cAMP by inhibition of adenylate cyclase, while CBD binds very weakly to CB1, behaving as a non-competitive negative allosteric modulator that reduces the efficacy and potency of Δ^9^-THC [160]. CB1 receptors contribute to pain relief and relaxation through the induction of endorphin release, while the activity of CBD at the 5-HT1A serotonin receptors may explain its neuroprotective, antidepressive, and anxiolytic effects [150].

CB2 receptors are found primarily on cells of the immune system including T cells [125] and microglia [169] and are also expressed in the hippocampus, cerebral cortex, cerebellum, globus pallidus, nucleus accumbens, and dorsal striatum, as well as in the PNS, dendritic cells [124], GI tract, cardiovascular system, liver, bone, adipose tissue, and reproductive system [159,160,161,170,171,172,173,174,175]. Activation of CB2 receptors mediates neuroprotection [124], reparative activation, and maintenance of bone mass. CBD and Δ^9^-THC act as partial agonists at CB2, mediating immunosuppression [176], decreased leukocyte rolling and adhesion [124], suppression of T-cell proliferation, and induction of T-cell apoptosis, which could stimulate target cell survival, migration, and growth, through numerous signaling pathways involving inhibition of adenylyl cyclase, decreased cAMP production, decreased protein kinase (PKA), regulation of A-type potassium channels, activation of Akt/protein kinase B, regulation of the MAPK cascade, inhibition of calcium channels, stimulation of the de novo synthesis of ceramide, and potential activation of β-arrestin-specific signaling [160,177].

A third receptor that is activated by cannabinoids, and that exhibits affinity with both CBD and Δ^9^-THC, is the G protein-coupled receptor 55 (GPR55), which is also activated by non-cannabinoid lipids [178,179,180]. GPR55 expression has been described in the striatum (putamen, caudate nucleus), hippocampus, hypothalamus, frontal cortex, monocytes, and NK cells. GPR55 activates the Gαq/12 and Gα13 G proteins and increases intracellular Ca^2^^+^, ERK phosphorylation, protein RhoA activation, and hippocampal release of glutamate, mediating procedural memory modulation, motor coordination, and anxiety-like behavior [180], and having a proinflammatory role in innate immunity [71]. The cannabinoid receptors GPCR119 and GPCR18 are also alternative cannabinoid receptors, as are transient receptor potential cation channels (TRPVs) and nuclear peroxisome proliferator-activated receptors (PPARs) [160,181,182].

In addition to neuroprotective effects, cannabinoid treatment in vitro and in vivo has antiseizure, antiemetic, anti-inflammatory, and antitumor effects which include antiproliferative, proapoptotic, autophagic, antiadhesion, antimigration, and antiangiogenic mechanisms affecting cancer cells, cell lines, human tumor xenografts, and animal cancer models [160,177]. 

The question of interest in MS is whether cannabinoid pharmacotherapy could go beyond immunomodulation to achieve the therapeutic goals yet to be fulfilled of restorative remyelination and repair within the CNS. 

The suggested neuroprotective/regenerative/immunosuppressive role for endocannabinoids in MS [183,184] was further supported by evidence that levels of several endocannabinoids detected in the CSF were lower in patients with RRMS and SPMS as compared to controls and were lowest in SPMS but increased, yet remained below normal, during relapses, and in patients with MRI gadolinium-enhancing (Gd+) lesions [185]. In post-mortem brain sections from MS patients, CB1 receptors were expressed by cortical neurons, oligodendrocytes, oligodendrocyte precursors, macrophages, and T lymphocytes, while CB2 receptors were present in T cells, astrocytes, microglia within “active” lesions, and microglia in the periphery of “mixed active/inactive” (chronic/active) lesions [186]. 

In EAE, increased endocannabinoid levels in the brain, cerebellum, and plasma of C57BL/6 mice treated with a selective endocannabinoid reuptake inhibitor, WOBE437, correlated with significant muscle relaxation, decreased neuroinflammatory infiltration, and lower microglial proliferation [187]. Dynamic transcriptional deregulation in the endocannabinoid system during EAE results in an early shift toward enhanced 2-AG-mediated CB1 receptor activation in astrocytes that is followed by deficits at later stages of the disease [188].

The following is a discussion of a variety of cellular and molecular mechanisms that may be targeted using cannabinoids to favorably impact the long-term clinicopathologic outcomes in MS by contributing to five major functional goals.

### 4.3. Mechanisms That Would Inhibit Innate Immune Assaults on CNS White Matter and Cortex

Recent studies have provided evidence implicating lysophosphatidic acid (LPA) signaling through its G protein-coupled receptor, LPA1, as a mechanism of macrophage activation that correlates with the onset of relapses and greater disease severity in both EAE and MS [189]. Interactions between the endocannabinoid system and the LPA system in the mouse brain have been identified in studies demonstrating upregulation of LP1 receptor activity in CB1 knockout mice [190]. Cannabinoids have also been shown to promote a reparative activation state of microglia and macrophages, diminishing the reactivity and the number of microglia in Theiler’s virus-induced demyelination disease [128]. RNA sequencing analysis has revealed that oral CBD treatment of EAE mice can reduce the expression of CXCL9, CXCL10, and IL-1β, leading to decreased macrophage infiltration into the CNS, and also induce myeloid-derived suppressor cells (MDSCs) [191].

### 4.4. Mechanisms That Would Downregulate Antigen-Specific Adaptive Immune Responses within the CNS

In vivo intravital microscopy via a cranial window has revealed that cannabinoid agonist WIN 55212-2 treatment attenuates leukocyte rolling and adhesion to endothelial cells in EAE, suggesting that cannabinoids could interfere with T-cell migration into the CNS through CB2 receptor-mediated downregulation of adhesion molecules [192]. 

The inhibition of autoreactive T-cell proliferation following treatment with cannabinoids is believed to be the result of the increased mobilization, recruitment, and enhanced potency of MDSCs in response to cannabinoid receptor activation, leading to increased apoptotic cell death of CD4+ T cells in the brain [85,191,193]. Other mechanisms that may contribute to cannabinoid inhibition of T-cell responses are their ability to induce IL-10 and TGF-β, inhibit IL-2, and Th17/Th1 differentiation, influence the mechanism of antigen presentation, and impede adhesion by downregulation of migration-related signaling pathways [106,123,194]. 

Other approaches that have shown promise in targeting antigen-specific autoreactive responses in EAE include the optimization of antigen-specific cell therapies. For example, the presence of interferon beta (IFN-beta) can enhance the suppressive effects of MOG_35–55_-loaded, vitamin D3-tolerogenic dendritic cells (VitD3-tolIDC-MOG) on activated T cells, resulting in shifts towards anti-inflammatory Th2 profiles and amelioration of clinical signs of EAE [195]. Through activation of the CB1 and PPARα receptors, cannabinoids promote human tolerogenic dendritic cells, with the capacity to prime FOXP3^+^ Tregs, through mechanisms involving the induction of functional autophagy and activation of AMPK, with subsequent increased mitochondrial oxidative phosphorylation [196].

### 4.5. Mechanisms That Would Promote Oligodendrocyte Survival

Treatment of human oligodendrocytes in culture with CBD has been found to upregulate the expression of proteins involved in myelination including microtubule-associated proteins, Rho GTPase-activating proteins, transient receptor potential channels, and the voltage-dependent T-type alpha 1H subunit of the calcium channel [197]. Assessment of white matter integrity by diffusion tensor imaging and transmission electron microscopy has revealed that treatment of a traumatic brain injury rat model with CB2 agonists increased levels of myelin basic protein, neurofilament heavy chain (NF200), oligodendrocyte precursor cells, mature oligodendrocytes, and anatomic preservation of myelinated axons [198]. CB2-mediated facilitation of oligodendrocyte survival involved decreases in the phosphorylation of the protein kinase R (PKR)-like endoplasmic reticulum (ER) kinase (PERK), eIF2α, ATF4, and GADD34 (Growth Arrest and DNA Damage-inducible protein) signaling pathway in microglia [198]. In EAE, CB2-mediated inhibition of demyelination manifests as preservation of myelin staining in CNS tissues [123]. In earlier studies, treatment with cannabinoid agonists prevented the loss of mature oligodendrocytes and the loss of myelin in the spinal cords of EAE mice, an effect that was blocked by CB1 antagonists [199]. 

### 4.6. Mechanisms That Would Provide Neuroprotection and Stimulate Axonal Regeneration

Axonal loss, as measured either by axonal thinning or by the density of NF200-positive axons in the spinal cord, was prevented by the treatment of EAE mice with cannabinoid agonists [123,199]. Treatment with a CB1/CB2 receptor agonist has been shown to decrease TNF-α-induced apoptosis of hippocampal neurons, a pro-survival effect that is abrogated by blockade of ERK1/2 [84]. Conversely, treatment with CB2 agonists enhances TNF-α-induced neuronal cell death, a proapoptotic effect that is abrogated by p38 MAPK activation [200]. 

Evidence suggests that the neuroprotective mechanisms of CB1 activation, demonstrated in EAE, against excitotoxic neurodegeneration involve countering the effects of IL-1β on glutamate release from presynaptic nerve terminals, in addition to reversing the enhancing effects of TNF-α on postsynaptic glutamate receptor expression and function [180,201,202]. This abrogation of glutamate-induced cell injury could have therapeutic effects in MS since excess glutamate has been shown to play a role in the pathogenesis of MS [203].

### 4.7. Mechanisms That Would Support CNS Remyelination

In addition to oligodendroglial loss as a cause of demyelination, failure of remyelination in MS lesions is attributed to extrinsic immune factors in the inflammatory environment, whereby oligodendroglial progenitor cell differentiation is impaired in the presence of CD4+ T cells, interferon-gamma, and the M1-polarized microglia cytokines IL-6, CXCL10, and TNFα, while remyelination in mixed lesions is decreased in the presence of greater proportions of TMEM119+ microglia and inducible nitric oxide synthase (iNOS)+ myeloid cells [204,205]. Failure of remyelination is also attributed to the inhibition of oligodendrogenesis in MS lesions by bone morphogenetic proteins (BMPs) through the BMPRII and pSMAD1/5/8 pathways [206]. Quantifying any effects of cannabinoid treatment on these cytokine, chemokine, and BMP signaling pathways would shed light on their potential role in reversing the failure to remyelinate in MS.

Remyelination can be enhanced by microglial necroptosis to eliminate populations of proinflammatory (iNOS+ TNFα+ CCL2+) microglia, followed by the proliferation of residual CNS microglia to repopulate the CNS with proregenerative (Arg-1+ CD206+ IGF-1+) microglial populations [207]. The differentiation of oligodendrocyte precursor cells (OPCs) is promoted by the pro-differentiation factors Olig2, Sox10, Myelin regulatory factor (Myrf), miR-219, and miR-338 and a type 1 transmembrane protein encoded by the CNS myelin gene, TMEM10, also called Opalin, to activate the expression of the 2′,3′-Cyclic-Nucleotide 3′-Phosphodiesterase (CNP), UDP-galactose:ceramide galactosyl transferase (CGT), Myelin Associated Glycoprotein (MAG), Proteolipid Protein 1 (PLP), and Myelin Basic Protein (MBP) genes [208]. In mice with cuprizone (Cpz) diet-induced demyelination, intraperitoneal treatment with 3 mg·kg^−1^ Δ^9^-THC for 5 days increased myelin ultrastructure thickness, and expression levels of MAG, myelin oligodendrocyte glycoprotein, and MBP, improved motor function recovery, decreased anxiety-like behaviors, and increased densities of differentiating progenitor CC1+ oligodendrocytes [126]. Treatment with Δ^9^-THC also promoted axonal remyelination in organotypic cerebellar cultures [126]. CB1 receptors regulate the mTORC_1_ signaling pathway during oligodendrocyte development [209], and the remyelination effects could be abrogated by both mTORC_1_ blockade and CB1 receptor-selective antagonism [126].

### 4.8. Challenges and Opportunities of Cannabinoid Research

A questionnaire survey of 1513 New England dispensary members in 2017 revealed that regular use of medical marijuana reduced reliance on opioids, benzodiazepines, antidepressants, alcohol, and anti-anxiety, migraine, and sleep medications in significant proportions of the population [210]. Analysis of patient-reported outcome measures in 312 patients from the UK medical cannabis registry revealed statistically significant improvements at 6 months in the Generalized Anxiety Disorder Scale score (GAD-7, *p* < 0.001), the EuroQol Group EQ-5D-5L index value (*p* < 0.001), the EQ-5D Visual Analog Scale score (VAS, *p* < 0.012), and the Sleep Quality Scale (SQS, *p* < 0.001) score [211]. The most common adverse effects reported were nausea (3.8%), dry mouth (3.2%), dizziness (2.2%), and somnolence (2.2%) [211]. Challenges faced with the use of medical marijuana include a narrow therapeutic window, as well as deactivation of cytochrome P450 and other liver enzymes, thus altering the metabolism of a wide range of compounds. Tolerability thresholds vary between individuals and have been an important reason for withdrawal from the studies. Hence, there are occasional difficulties in attaining intention-to-treat primary outcomes, limiting the final analyses to data collected from smaller-sized samples of participants who have the capacity to complete their regimens per protocol. Another challenge is related to finding heterogeneity that separates cannabinoid-treated populations into “responders” and “non-responders”.

## 5. Conclusions

Medical marijuana studies conducted between 2007 and 2021 have demonstrated, with a moderate certainty of evidence, that add-on therapy with 1:1 CBD/THC cannabinoid oromucosal spray mixtures is effective within a narrow therapeutic window to modestly improve primarily subjective measures of spasticity, pain, and bladder- and sleep-related quality of life in responders within weeks of starting treatment. Some benefits are maintained beyond 6 months to 12 months, but some effects may wane with prolonged use. Further studies are warranted to further investigate the long-term effects of cannabinoid adjunctive therapy on MS disease progression and accrual of disability, and to elucidate the exact cellular and molecular mechanisms responsible for inhibiting neuroinflammation, enhancing neuroprotection, and promoting remyelination in the CNS.

## Figures and Tables

**Table 1:** Effects of cannabinoids on spasticity in multiple sclerosis.

Study	Year	Findings	Formulations
D’hooghe et al. *Retrospective Cohort Study* [105]	2021	A total of 276 patients with MS for at least 6 months and spasticity for at least 3 months were included in a retrospective cohort study from 8 MS centers in Belgium. In 238 evaluable patients, 73% reported ≥20% decrease in spasticity NRS scores, experiencing reduced severity within 4 weeks. A total of 50% reported ≥30% improvement. Mean spasticity NRS scores improved from 8.1 (±1.08) at baseline to 5.2 (±1.85) at week 4, and 4.6 (±1.69) at week 8. A total of 80 patients discontinued within 8 weeks. By week 12, 171 patients reported a clinically meaningful response with a mean NRS score of 4.1 (±1.78) at week 12. More than 60% of the patients with MS who started add-on treatment with cannabinoid oromucosal spray reported a clinically relevant symptomatic effect and continued treatment after 12 weeks, and some continued to gain improved spasticity outcomes after 12 weeks. NRS improvement was maintained with mean scores of 4.3 (±1.77) at 6 months (n = 180) and 4.0 (±1.92) at 12 months (n = 113) [105].	Sativex^®^ oromucosal spray at 6 sprays/day. Sativex^®^ was the add-on therapy to, at minimum, oral baclofen.
Sorosina et al. *Observational Clinical Study* [106]	2016 2018	A total of 93 nabiximols-treated MS patients across Italy were enrolled in an observational study. Whole blood was collected at baseline, 4 weeks (n = 93), and 14 weeks (n = 33, n = 19 responders and n = 14 non-responders) and analyzed by whole-genome microarray-based transcriptome profiling using Illumina^®^ technology. Network analysis using the STRING interactome resource allowed comparisons between high responders and non-responders. Improvement in mean spasticity NRS scores of −2.9 at 4 weeks and −3.6 at 14 weeks in high responders to treatment (n = 19), as compared to +0.1 mean change in non-responders, as well as improvement in pain (mean change −3.6) scores, was associated with upregulation of ribosome pathway genes and downregulation of genes related to cell motility/migration, and immune and nervous systems including a genetic signature of 22 genes that differentiated responders from non-responders (*p* < 0.05) [106].	Nabiximols (Sativex^®^) oromucosal spray 7 ± 2 (in responders) and 8 ± 3 (in non-responders) for 4 weeks. A total of 60 (81%) patients were treated with <10 mg/kg/d of CBD. Patients receiving other disease-modifying drugs were excluded.
Flachenecker et al. *Prospective, Multicenter Cohort Study* [107]	2014a	A total of 276 patients across Germany with moderate to severe RRMS, SPMS, PPMS, and progressive and remitting MS were enrolled in an observational, prospective, multicenter study (MOVE2). The distribution of EDSS was ≤4.0 (17%), 4.5–6.5 (51%), and ≥7.0 (32%). After 1 month, nabiximols treatment provided relief of resistant MS in 74.6%, with a 12.3% reduction in mean spasticity NRS-11 score from 6.1 ± 1.7 to 5.2 ± 1.9, 41.7% experienced a ≥20% response, and 25.5% experienced ≥30% improvement from baseline (*p* < 0.0001; n = 216). After 3 months, 58.7% experienced ≥20% response, and 40.0% experienced ≥30% improvement from baseline (*p* < 0.0001; n = 95), reaching a mean NRS score of 4.7. Additionally, mean scores on the MSQoL-54 physical health composite (n = 47) improved by 25% from 39.2 ± 15.2 to 45.0 ± 15.0 (*p* = 0.0003), and on the MSQoL-54 mental health composite (n = 55), mean scores improved by 19% from 47.0 ± 17.1 to 53.1 ± 17.1 (*p* = 0.0012) [107].	Nabiximols (THC/CBD 1:1, Sativex^®^) delivered using a pump action oromucosal spray, mean of 6.9 ± 2.8 sprays/day. A total of 89.5% of patients tried other antispastic drugs. A total of 72.8% of patients used nabiximol as an add-on to baclofen (50.0%), tolperisone (16.3%), tizanidine (13.8%), and gabapentin (9.1%). A total of 27.2% of patients had no concomitant antispastic medication.
Flachenecker et al. *Prospective, Multicenter Cohort Study* [108]	2014b	A total of 51 MS patients participated in the 12-month prolongation of the prospective, multicenter cohort study in Germany (MOVE2 study). After 12 months of treatment with nabiximols, 52.9% (n = 27) had at least a 20% reduction in spasticity NRS score (*p* = 0.0004), and 41.2% (n = 21) had at least a reduction of 30% in spasticity NRS score (*p* = 0.0038). The mean NRS spasticity score decreased from 6.2 ± 1.8 to 4.6 ± 2.1 (*p* < 0.0001, n = 51) [108].	Nabiximols (THC/CBD 1:1, Sativex^®^) delivered using a pump action oromucosal spray, mean of 6.9 ± 2.8 sprays/day.
Koehler et al. *Retrospective Cohort Study* [109]	2016	A total of 166 patients, 80% with secondary progressive MS, received treatment with THC/CBD spray (add-on n = 95; monotherapy n = 25) for a mean of 9 months. A clinical chart review was conducted over a 15-month period. The mean spasticity NRS score decreased from 7.0 to 3.0 in responders within 10 days, representing a 57% reduction [109].	THC/CBD oromucosal spray in a 1:1 ratio. Mean 4.0 ± 2.6 sprays/day for add-on therapy and mean 3.0 ± 2.6 sprays/day for monotherapy. Other oral antispasticity medications used included baclofen, gabapentin, pregabalin, tolperisone, tetrazepam, and dantrolene.
Paolicelli et al. *Prospective Cohort Study* [110]	2015	See Table 3.	See Table 3.
Novotna et al. *Randomized, Double-Blind (Phase B), Controlled Trial* [111]	2011	A total of 241 MS patients with a mean duration in excess of 12 years, and a mean spasticity duration in excess of 7 years, were enrolled in a multicenter (51 sites in the United Kingdom, Italy, Poland, the Czech Republic), double-blind (Phase B), randomized, placebo-controlled, parallel-group study. After 12 weeks, the estimated difference between the nabiximol treatment (n = 124) and placebo (n = 117) groups in mean spasticity scores was 0.84 points (95% CI: −1.29 to −0.40) (*p* = 0.0002). The proportion of patients with at least a 30% improvement in spasticity in the active treatment group was significantly higher than in the placebo group (74% vs. 51%: odds ratio 2.73 (95% CI 1.59 to 4.69, *p* = 0.0003)) [111].	Nabiximols add-on therapy delivered using a pump action oromucosal spray with each 100 μL actuation yielding 2.7 mg THC and 2.5 mg CBD. Subjects up-titrated to their optimal dose according to a predefined escalation scheme. Concomitant antispastic medications used were adamantane derivatives, benzodiazepine-related derivatives, dantrolene, antiepileptics, baclofen, tizanidine, and tolperisone.
Collin et al. *Randomized, Double-Blind, Placebo-Controlled Parallel-Group Study* [112]	2010	A total of 337 patients with MS spasticity not fully relieved with antispasticity therapy were enrolled in a 15-week (1-week baseline and 14-week treatment period), multicenter (15 centers in the UK and 8 in the Czech Republic), double-blind, randomized, placebo-controlled, parallel-group study. Sativex^®^-treated population (n = 150) showed a significant reduction in spasticity NRS scores with ≥30% improvement from baseline (−1.3 points) vs. placebo (n = 55, −0.8 points) (*p* = 0.035). In subjects who achieved ≥30% response, 98, 94, and 73% reported improvements of 10, 20, and 30%, respectively, at least once during the first 4 weeks of treatment [112].	Sativex^®^ pump action oromucosal spray. Each 100 mL actuation (maximum 8 in a 3 h period and 24 in a 24 h period) of active medication delivered a dose containing 2.7 mg THC and 2.5 mg CBD, self-titrated by patients to optimal dose. Concomitant medications included baclofen, azathioprine, and methylprednisolone.
Collin et al. *Randomized, Double-Blind, Placebo-Controlled Clinical Trial* [113]	2007	A total of 189 patients diagnosed with MS for 12–13 years from the UK were enrolled in a randomized, double-blind, placebo-controlled clinical trial. After 6 weeks, a greater proportion of the Sativex^®^-treated group (40%, n = 48) achieved a >30% reduction in spasticity as compared to the placebo group (22%, n = 14), a statistically significant difference (*p* = 0.014). Treatment group (n = 124) showed NRS reduction of 1.18 points from baseline, while placebo group (n = 65) showed reduction of 0.63 points from baseline. The estimated difference in mean spasticity score between the Sativex^®^ treatment and placebo groups was 0.52 points (*p* = 0.048) [113].	Sativex^®^ oromucosal spray with each 100 μL actuation yielding 2.7 mg of Δ^9^-THC and 2.5 mg of CBD. Subjects up-titrated their daily dose over 2 weeks to a maximum of 48 sprays per day. Concomitant antispasticity medications were used and not listed.

**Table 2:** Effects of cannabinoids on pain intensity in multiple sclerosis.

Author	Year	Findings	Formulations
Turri et al. *Observational Clinical Study* [114]	2018	A total of 28 MS patients were enrolled in an observational study to assess the effects of Sativex^®^ oromucosal spray on pain. Of the 19 patients who completed the study, 8 presented with neuropathic pain, 6 had nociceptive pain, and 5 reported mixed pain. After receiving Sativex^®^ for 1 month, the subjects reported a reduction in mean pain scores from 6.61 to 3.55 on the 11-NRS (*p* < 0.0001) [114].	Sativex^®^ oromucosal spray daily. All patients gradually increased their dose of oromucosal spray of Sativex^®^ until they achieved a satisfactory number of administrations per day (mean puffs/day 6.9 ± 1.9, range 4–11).
Russo et al. *Observational Clinical Study* [115]	2016	A total of 20 MS patients in Italy were enrolled in an observational study that assessed clinical and neurophysiologic parameters before and after 4 weeks of treatment with Sativex^®^. Half of the patients had neuropathic pain. After one month of drug administration, those with neuropathic pain (n = 10) reported a reduction in pain on the VAS rating scale from 7 ± 1 down to 1 ± 1 (*p* = 0.001), and an improvement in quality of life on the MSQoL (*p* = 0.03) [115].	Sativex^®^ daily in a pump action sublingual spray, mean 8 sprays/day. THC (27 mg/mL) and CBD (25 mg/mL), with ethanol/propylene glycol (50:50) excipient. A pump delivers 100 mL of spray, containing THC 2.7 mg and CBD 2.5 mg. Concomitant baclofen.
Sorosina et al. *Observational Clinical Study* [106]	2016 2018	See Table 1.	See Table 1.
Paolicelli et al. *Prospective Cohort Study* [110]	2015	See Table 3.	See Table 3.
Langford et al. *Randomized, Double-Blind, Placebo-Controlled, Multicenter Clinical Trial* [116]	2013	A total of 339 MS patients were enrolled in a double-blind, placebo-controlled, multicenter (33 sites in the UK, Canada, Spain, France, the Czech Republic) study to assess the effects of Sativex^®^ oromucosal spray on pain in patients who had failed to gain adequate analgesia from existing medication. A total of 58 patients entered Phase B which consisted of a 2-week re-titration period and a 12-week stable dose phase with THC/CBD spray, with an additional 4-week randomized withdrawal phase. A statistically significant treatment difference in favor of THC/CBD spray was observed at week 10 (*p* = 0.046). During the randomized withdrawal phase, the time to treatment failure was 57% of patients receiving placebo vs. 24% of patients receiving THC/CBD spray, a statistically significant difference (*p* = 0.04), showing a mean change from baseline in pain NRS (−0.79, *p* = 0.028) and sleep quality NRS (0.99, *p* = 0.015) scores [116].	Sativex ^®^ oromucosal spray as add-on treatment, each 100 μL actuation yielding THC 2.5 mg and CBD 2.5 mg. Concomitant analgesic medications included NSAIDs, anticonvulsant, tricyclic antidepressants, and opioids.

**Table 3:** Effects of cannabinoids on lower urinary tract dysfunctions in multiple sclerosis.

Author	Year	Findings	Formulations
Maniscalco et al. *Pilot Prospective Study* [117]	2018	A total of 15 MS patients with overactive bladder and spasticity NRS-11 ≥ 4 were enrolled in a pilot prospective study to assess the effects of Sativex^®^ on resistant MS bladder symptoms. After 4 weeks of treatment with Sativex^®^, the median spasticity NRS improved from 8 to 6 (*p* < 0.001), the median OABSS (overactive bladder symptom score) total score decreased from 17 to 12 (*p* = 0.001), and the median PVR decreased from 80 mL to 30 mL (*p* = 0.016) [117].	Nabiximols (Sativex^®^) THC/CBD oromucosal spray with a mean puffs per day of 3.8 ± 1.02. Concomitant medications used were interferon, teriflunomide, glatiramer acetate, diethyl fumarate, and natalizumab.
Paolicelli et al. *Prospective Cohort Study* [110]	2015	A total of 102 MS patients (58% secondary progressive, 10% primary progressive, and 25% remitting-relapsing) enrolled in a prospective cohort study and administered THC/CBD spray as add-on therapy. After 1 month, there was a reduction in the mean spasticity NRS score from 8.7 ± 1.3 to 6.2 ± 1.8 with stabilization at this level at 3-, 6-, and 12-month follow-up (*p* < 0.001). A total of 57% of subjects who had pain refractory to gabapentinoids (n = 33) reported improvement in pain NRS scores at the first month (6.1 ± 2.5 vs. 3.4 ± 2) that remained stable at the sixth-month follow-up (*p* = 0.011). Bladder dysfunction IPSS score decreased from 15 ± 8.2 to 9.9 ± 7.4 at the first month (*p* = 0.011) and 9.3 ± 9.6 at the 6-month (*p* = 0.008) follow-up. Bladder-related QoL score decreased from 2.1 ± 0.5 to 1.5 ± 0.6 at the first month (*p* = 0.001) and 1.8 ± 0.7 at the 6-month (*p* = 0.041) follow-up [110].	Sativex^®^ THC/CBD oromucosal spray with an average of 6.5 ± 1.6 sprays each day. Concomitant drugs used were interferon-β, glatiramer acetate, azathioprine, fingolimod, and natalizumab.
Kavia et al. *Randomized, Double-Blind, Placebo-Controlled, Parallel-Group Trial* [118]	2010	A total of 118 (56 Sativex^®^, 62 placebo) MS patients with overactive bladder symptoms refractory to other treatments completed per protocol a randomized, double-blind, placebo-controlled, parallel-group trial. At 8 weeks after treatment, the difference between the treatment and control groups in adjusted mean change was 0.85 in total number of voids per 24 h, 0.28 in the number of nocturia episodes per day and the number of daytime voids, and 0.76 in the number of void urgency episodes per day in favor of the Sativex-treated group (n = 60) as compared to the placebo group (n = 64), and the differences were statistically significant at the level of *p* < 0.05. The difference in adjusted mean change was 1.16 for overall bladder condition, with OBS NRS score showing improvement to a greater extent in the Sativex^®^-treated group (n = 61) as compared to the placebo group (n = 66) (*p* = 0.001) [118].	Nabiximols (Sativex^®^) add-on treatment, pump action oromucosal spray 2.7 mg THC:2.5 mg CBD. Maximum permitted dose 8 puffs in any 3 h period and 48 puffs in any 24 h period. Concomitant anticholinergic medication.

**Table 4:** Effects of cannabinoids on sleep disturbance in multiple sclerosis.

Author	Year	Findings	Formulations
Braley et al. *Cross-Sectional Survey-Based Study* [119]	2020	Of 427 individuals who reported using cannabis in the past year either recreationally or for medical reasons through a University of Michigan national survey of MS patients, 70% reported benefits for pain, 56% reported benefits for sleep concerns, and 49% reported benefits for spasticity. The self-reported benefits in ability to fall asleep were significant (n = 180, *p* < 0.001) in the sub-groups who reported using low THC/CBD (n = 6), high THC/CBD (n = 27), CBD monotherapy (n = 46), and THC monotherapy (n = 15). The sleep benefits strongly correlated with relief in pain that interferes with sleep (r = 0.65, *p* < 0.0001). For those who expressed a preference for specific THC/CBD ratios, CBD-predominant formulations were favored [119].	Self-reported formulations used daily for a year included cannabis, smoking, edibles, vaping, topical lotions/patches, and capsules, classified into the following 4 categories: low THC/low CBD, high THC/high CBD, CBD monotherapy or predominant therapy, and THC monotherapy or predominant therapy.
Flachenecker et al. *Prospective, Multicenter Cohort Study* [108]	2014b	A total of 50 MS patients participated in the 12-month prolongation of the prospective, multicenter cohort study in Germany (MOVE2 study). After 12 months of treatment with nabiximols, the mean sleep NRS score decreased from 5.1 ± 2.9 down to 3.2 ± 2.5 points (*p* < 0.001) [108].	Nabiximols (THC/CBD 1:1, Sativex^®^) delivered using a pump action oromucosal spray, mean of 6.9 ± 2.8 sprays/day.
Langford et al. *Randomized, Double-Blind, Placebo-Controlled, Multicenter Clinical Trial* [116]	2013	See Table 3.	See Table 3.

**Table 5:** Effects of cannabinoids on inflammation and spasticity in EAE animal model of MS.

Author	Year	Findings	Formulations
Nichols et al. *Observational Experimental* *Animal Study* [87]	2021	EAE was induced in C57BL/6 mice, and oral CBD treatment was started 5 days later. At day 10, there was a significant decrease in the proportion of MOG_35–55_ specific, IFN-γ producing CD8^+^ T cells in the spleen (*p* < 0.05). At day 18, clinical EAE scores (on a scale of 1–5) were significantly reduced (*p* < 0.05). Trends were noted for moderate reductions in the number of T cells (*p* = 0.098) and the size of infiltrated and inflammatory lesions (*p* = 0.13) within spinal cord tissues [87].	Oral CBD 75 mg/kg for 5 days after initiation of EAE.
Elliott et al. *Observational Experimental* *Animal Study* [120]	2018	C57BL/6 mice with MOG_35–55_ peptide-induced EAE treated with CBD exhibited delayed onset and decreased severity of clinical EAE (maximum score of 2.2 ± 0.16). The CBD treatment significantly reduced the proportions of CD4^+^ and CD8^+^ T-cell infiltrates in brain and spinal cord tissues and reduced levels of IL-17 and IFN-γ inflammatory cytokines as compared to controls (*p* < 0.0001) [120].	CBD (20 mg/kg; 16% DMSO/PBS) was injected daily intraperitoneally, starting at day 9 through day 25.
González-García et al. *Observational Experimental* *Animal Study* [121]	2017	C57BL/6J mice with adoptively transferred EAE, induced by MOG_35–55_-specific T cells, were treated daily with 50 mg/kg of CBD intravenously. CBD markedly improved the clinical signs of EAE and reduced infiltration, demyelination, and axonal damage (*p* < 0.01). The CBD-mediated decrease in the viability of encephalitogenic T cells involved ROS generation, apoptosis, and a decrease in IL-6 production [121].	CBD was administered intravenously at 50 mg/kg daily.
Kozela et al. *Observational Experimental Study* [122]	2016	Encephalitogenic TMOG cells were stimulated with MOG_35–55_ in the presence of spleen-derived antigen-presenting cells (APCs) with or without CBD. Gene expression profiling showed that the CBD treatment inhibited transcription of proinflammatory cytokines, cytokine receptors, MOG_35–55_-induced TMOG cell proliferation and Th17 activity, transcription factors, and TNF superfamily signaling molecules, while increasing antiproliferative interferon-dependent transcripts (*p* < 0.005). Furthermore, CBD enhanced the transcription of oxidative stress modulation [122].	Incubation of T cells in vitro for 8 h with 5 μM CBD.
Kong et al. *Observational Experimental Study* [123]	2014	MOG_35–55_ EAE C57BL/6 mouse treatment with a selective CB2 receptor agonist, Gp1a, reduced severity and facilitated clinical recovery (*p* < 0.05), decreased accumulation of total mononuclear cells (*p* < 0.05), CD3^+^ and CD4^+^ T cells (*p* < 0.05), and Iba-1^+^ (activated) microglia/macrophages in CNS tissues, decreased production of INF-γ (*p* < 0.05) and IL-17 (*p* < 0.01) in splenocytes (in contrast to wildtype controls and CB2R knockout mice), decreased demyelination and axonal degeneration, decreased gene expression of chemokine receptors, chemokines, and adhesion molecules in vivo, and inhibited CD4^+^ Th1/Th17 differentiation of splenocytes in vitro (*p* < 0.05) [123].	Gp1a-selective CB2 receptor agonist (N-(piperidin-1-yl)- 1-(2,4-dichlorophenyl)-1,4-dihydro-6-methylindeno[1,2-c]pyra zole-3-carboxamide) 5 mg/kg via tail vein injection, twice per week starting either day 0 or day 7. Splenocytes cultured from transgenic EAE (MOG35–55-specific TCR) in either CB2 receptor (Cnr2) +/+ or +/− knockout mice on C57BL/6 background were treated with 5 and 10 μM Gp1a in vitro.
Hilliard et al. *Observational Experimental Study* [86]	2012	Active remitting-relapsing EAE was induced in adult Biozzi ABH mice by subcutaneous injection on days 0 and 7 of mouse spinal cord homogenate. Intravenous injections with either cannabinoids alone or baclofen alone or vehicle were administered to mice in post-relapse remission, 7–8 months after inoculation. Spasticity, “stiffness”, and measurements of force needed to bend hindlimb to full flexion were assessed 10 min after intravenous treatment. Low dose (5 mg/kg THC + 5 mg/kg CBD) caused a 20% reduction in hindlimb stiffness (*p* = 0.007). Higher dose (10 mg/kg THC + 10 mg/kg CBD) caused a 40% reduction in hindlimb stiffness (*p* = 0.002). Baclofen (5 mg/kg) also provided a 40% reduction in hindlimb stiffness; however, cannabinoid treatment appeared to be better tolerated [86].	A 0.1 mL solution containing a 1:1 ratio of THC/CBD was administered by tail vein injection of either low dose, 5 mg/kg, or high dose, 10 mg/kg. Positive control group received 5 mg/kg baclofen alone.
Kozela et al. *Observational Experimental Study* [85]	2011	Active EAE was induced in C57BL/6 mice by subcutaneous injections on day 1 and day 8 of 300 µg of MOG_35–55_ peptide. Mice were treated intravenously with either CBD (5 mg/kg) or vehicle on days 19, 20, and 21. The mean clinical EAE scores were lower in the CBD-treated group beginning on day 19 and remained significantly lower over the next 11 days, markedly delaying disease progression as compared to controls. On day 21, the mean EAE scores were 0.13 ± 0.09 in CBD-treated mice as compared to 1.03 ± 0.29 in controls (*p* < 0.05). Furthermore, treatment with CBD significantly reduced CD3+ T-cell infiltration (*p* < 0.0001), Iba-1^+^ microglial/macrophage presence (*p* < 0.001) and activation (*p* < 0.001), and axonal damage in the spinal cord as compared to controls [85].	CBD 5 mg/kg was administered intravenously.
Zhang et al. *Observational Experimental Study* [124]	2009	Treatment of mice in either chronic EAE (C57BL/6/MOG_35–55_), remitting-relapsing EAE (SJL/J/PLP_139–151_), or adoptively transferred EAE with selective CB2 agonist, O-1966, significantly reduced disease severity (*p* < 0.05), leukocyte rolling (*p* < 0.05), and adhesion to cerebral microvessels in vivo (*p* < 0.05) [124].	Selective CB2 agonist O-1966 administered at 1 mg/kg on day 7 and every 4th day thereafter up to day 28.
Maresz et al. *Observational Experimental Study* [125]	2007	Treatment with 25 mg/kg of THC significantly reduced disease severity in actively induced EAE in ABH. Induction of active EAE in homozygous CB2 receptor knockout mice resulted in increased disease severity. Using CB2^−/−^ encephalitogenic T cells for induction of adoptive EAE enhanced disease severity (*p* < 0.05), increased T-cell proliferation in the CNS (*p* < 0.05), decreased apoptosis (*p* < 0.05), and increased production of IL-2 and IFN-γ (*p* < 0.05), demonstrating that CB2 receptor expression by T cells was critical for controlling inflammation in EAE [125].	THC 25 mg/kg delivered by intraperitoneal injections on days 10–22.

**Table 6:** Effects of cannabinoids on inflammation and spasticity in TMEV-IDD and toxin-induced demyelination models.

Author	Year	Findings	Formulations
Aguado et al. *Observational Experimental Study* [126]	2021	Cuprizone-fed mice treated with 3 mg/kg of THC for 5 days after discontinuation of Cpz diet showed improved motor functional recovery (*p* = 0.05) in conjunction with increased axonal myelination in the corpus callosum (CC) by electron microscopy on day 5, enhanced levels of MAG, MOG, and MBP myelin-associated proteins in CC extract, and enhanced oligodendrocyte regeneration 5 and 10 days after start of THC treatment via CB1 receptor-mediated effects as compared to vehicle controls (*p* < 0.05). Addition of 1 μM THC for 4 days to cerebellar organotypic slice cultures that had been subjected to lysolecithin (LPC)-induced demyelination in vitro enhanced MBP+ areas by confocal microscopy as compared to vehicle controls (*p* < 0.05) [126].	THC 3 mg/kg delivered by intraperitoneal injection for 5 consecutive days starting at 24 h after Cpz removal from the diet of mice that had been fed a Cpz diet for 6 weeks. THC 1μM added for 4 days to cerebellar organotypic slice cultures starting 15–17 h after incubation with lysolecithin (lysophosphatidylcholine).
Tomas-Roig et al. *Observational Experimental Study* [127]	2020	Cuprizone-fed mice treated with 0.5 mg/Kg cannabinoid agonist WIN-55,212-2 showed greater numbers of myelinated axons in the corpus callosum at 3 weeks and 6 weeks (*p* < 0.05) as compared to untreated Cpz-fed mice. However, CNS myelination was impaired by treatment with 1 mg/Kg WIN-55,212-2 [127].	0.5 mg/Kg cannabinoid agonist WIN-55,212-2 delivered by intraperitoneal injections to mice being fed a Cpz diet for 3 to 6 weeks. 1 mg/Kg WIN-55,212-2 had deleterious effects on remyelination.
Mecha et al. *Observational Experimental Study* [128]	2018	TMEV-infected SJL/J mice treated with either 5 mg/kg of 2-AG or a reversible inhibitor of 2-AG hydrolysis intraperitoneally for 7 days reduced the total number of microglia in the cerebral cortex (*p* ≤ 0.001), diminished the percentage area of Iba-1^+^ reactive microglia (2-AG *p* ≤ 0.05; UCM *p* ≤ 0.001), and hampered the development of jellyfish-like morphology, by confocal microscopy, characteristic of microglial activation. Treatment effects were not seen in Sham mice. 2-AG decreased expression of NOS-II in microglia (*p* ≤ 0.01), IL-1β (*p* ≤ 0.01), TNF-α, ICAM-1, chemokines (*p* ≤ 0.05), inflammatory infiltrates (*p* ≤ 0.001), and numbers of cells in lymph nodes (*p* ≤ 0.001); increased anti-inflammatory IL-10 (*p* ≤ 0.05), antiviral IFN-γ (*p* ≤ 0.05), proapoptotic bax and caspase 9 (*p* ≤ 0.05), numbers of cells in the spleen (*p* ≤ 0.001), numbers of immunosuppressive Arg-1^+^/CD11b^+^ MDSCs (*p* ≤ 0.05), and numbers of apoptotic CD4^+^ T cells in the brain (*p* ≤ 0.05); and improved long-term motor function (*p* ≤ 0.05). The AG-2 effects were mediated by the CB2 receptor [128].	Treatment of 5 mg/kg of endocannabinoid 2-AG or reversible inhibitor of 2-AG degradation UCM-03025 delivered by intraperitoneal injections daily for 7 days.
Arevalo-Martin et al. *Observational Experimental Study* [129]	2012	Treatment of TMEV-infected SJL/J mice with CB1/CB2 agonist WIN-55,212-2 for 10 days restored self-tolerance, decreased delayed-type hypersensitivity responses to myelin PLP_139–151_ peptide ~60 days after infection (dpi) (*p* < 0.001), induced long-lasting motor function recovery on rotarod, and vertical and horizontal activity measures (*p* < 0.001), decreased the activation of CD4^+^CD25^+^Foxp3^−^ T cells (*p* < 0.05), and increased regulatory CD4^+^CD25^+^Foxp3^+^ T cells (*p* < 0.01) in the CNS. Treatment reduced expression of IL-6 and CXCL1 in the CNS (*p* < 0.01) and upregulated production of IL-5, MCP-1, and IFN-γ (*p* < 0.05), and IL-9 and VEGF (*p* < 0.01) at 110 dpi as compared to vehicle-treated controls. Splenocytes transferred from WIN-treated mice at the onset of TMEV-IDD (30 dpi) inhibited the autoimmune inflammatory DTH response to PLP_139–151_ peptide 50 days later (80 dpi) and improved performance on motor function tests. Cyclophosphamide repressed WIN-induced self-tolerance and overrode WIN-induced recovery [129].	CB1/CB2 agonist WIN-55,212-2 by daily intraperitoneal injection; doses were progressively increased 2.5 mg/kg on days 1–3, 3.75 mg/kg on days 4–6, and 5 mg/kg on days 7–10 to avoid potential habituation.
Mestre et al. *Observational Experimental Study* [130]	2009	TMEV-IDD mouse treatment with 1.5 mg/kg CB1/CB2 receptor agonist WIN-55,212-2 for 3 days restricted CD11b^+^ microglial activation (*p* < 0.01), limited perivascular CD4^+^ T-cell infiltrates (*p* < 0.01), downregulated adhesion molecule-1 (ICAM-1, *p* < 0.01) and vascular cell adhesion molecule-1 (VCAM-1) in the brain endothelium, and improved motor coordination by rotarod and by horizontal and vertical activity by activity cage (*p* < 0.05) [130].	Treatment of 1.5 mg/kg CB1/CB2 agonist WIN55,212-2 administered for 3 consecutive days immediately after TMEV infection.

**Table 7:** Certainty of evidence.

Study	Risk of Bias ‡	Inconsistency	Indirectness	Imprecision	Dose–Response Relationship	Size of Effect	Confounding	GRADE
Spasticity								
D’hooghe [105]	Not serious	Not Serious	Not Serious	Serious	Very Serious ⁕	Not serious	Serious †	⊕⊕⊕〇 (Moderate)
Sorosina [106]	Not serious	Not Serious	Not Serious	Very Serious	Very Serious ⁕	Not Serious	Serious †	⊕⊕⊕〇 (Moderate)
Flachenecker [107]	Not serious	Not Serious	Not Serious	Serious	Very Serious ⁕	Serious	Serious †	⊕⊕〇〇 (Low)
Flachenecker [108]	Not serious	Not Serious	Not Serious	Serious	Very Serious ⁕	Serious	Serious †	⊕⊕〇〇 (Low)
Koehler [109]	Not Serious	Not Serious	Not Serious	Serious	Very Serious ⁕	Not Serious	Serious †	⊕⊕〇〇 (Low)
Paolicelli [110]	Not Serious	Not Serious	Not Serious	Serious	Very Serious ⁕	Not Serious	Serious †	⊕⊕⊕〇 (Moderate)
Novotna [111]	Not Serious	Not Serious	Not Serious	Serious	Very Serious ⁕	Serious	Serious †	⊕⊕⊕〇 (Moderate)
Collin [112]	Not Serious	Not Serious	Not Serious	Serious	Very Serious ⁕	Serious	Serious †	⊕⊕⊕〇 (Moderate)
Collin [113]	Not Serious	Not Serious	Not Serious	Serious	Very Serious ⁕	Serious	Serious †	⊕⊕⊕〇 (Moderate)
Pain								
Turri [114]	Serious	Not Serious	Not Serious	Serious	Very Serious ⁕	Not Serious	Unknown†	⊕⊕〇〇 (Low)
Russo [115]	Serious	Not Serious	Not Serious	Very Serious	Very Serious ⁕	Not Serious	Serious †	⊕〇〇〇 (Very Low)
Sorosina [106]	Not Serious	Not Serious	Not Serious	Very Serious	Very Serious ⁕	Not Serious	Serious †	⊕⊕〇〇 (Low)
Langford [116]	Not Serious	Not Serious	Not Serious	Serious	Not Serious	Serious	Serious †	⊕⊕⊕〇 (Moderate)
Paolicelli [110]	Not Serious	Not Serious	Not Serious	Serious	Very Serious ⁕	Not Serious	Serious †	⊕⊕⊕〇 (Moderate)
Lower Urinary Tract Dysfunction								
Maniscalco [117]	Not Serious	Not Serious	Not Serious	Very Serious	Very Serious ⁕	Serious	Serious †	⊕⊕〇〇 (Low)
Paolicelli [110]	Not Serious	Not Serious	Not Serious	Serious	Very Serious ⁕	Not Serious	Serious †	⊕⊕⊕〇 (Moderate)
Kavia [118]	Not Serious	Not Serious	Not Serious	Serious	Not Serious	Serious	Serious †	⊕⊕⊕〇 (Moderate)
Sleep Disturbance								
Braley [119]	Serious	Not Serious	Serious	Not serious	Serious	Unknown	Very Serious †	⊕〇〇〇 (Very Low)
Flachenecker [108]	Not serious	Not Serious	Not Serious	Serious	Very Serious ⁕	Serious	Serious †	⊕⊕〇〇 (Low)
Langford [116]	Not Serious	Not Serious	Not Serious	Serious	Not Serious	Serious	Serious †	⊕⊕⊕〇 (Moderate)

‡ attrition bias, performance bias; ⁕ narrow therapeutic window; † concomitant medications.

**Table 8:** Summary of findings.

Outcomes	Illustrative Comparative Risks (95% CI)		No. of Participants (Studies)	Quality of Evidence (GRADE)	Comments
	Assessment Risk (Usual Care/Control)	Corresponding Risk (Cannabinoid Treatment)			
Spasticity	Mean change in spasticity NRS score from baseline was −0.20.	Mean maximal change in spasticity NRS from baseline was −2.8 pts (min. −0.04, max. −7.4) between 12 wks and 12 mo. Mean of differences in spasticity NRS between treatment and control groups was −0.62 (range −0.5 to −0.83).	1582 (9 studies)	6 ⊕⊕⊕〇 (Moderate) 3 ⊕⊕〇〇 (Low)	Narrow therapeutic window. Confounding concomitant medication.
Pain		Mean change in pain score from baseline was −3.42 points (NRS), or −6 points (VAS) at 4 wks. Lower pain NRS by 0.79 pts at 12 wks.	573 (5 studies)	2 ⊕⊕⊕〇 (Moderate) 2 ⊕⊕〇〇 (Low) 1 ⊕〇〇〇 (Very Low)	Randomized withdrawal phase confirmed effect in clinical trial.
Lower Urinary Tract Dysfunction	-Adjusted mean change from baseline in total no. voids −0.9, urgency −1.12, nocturia episodes per day −0.24, and OBS NRS score −1.05 at 8 wks.	No benefit for urinary incontinence. Improved bladder dysfunction from baseline OABSS by 5 points, PVR −50 mL, IPSS by 5.1 pts at 4 wks, IPSS by 5.7 pts at 6 mo. Adjusted mean change from baseline total no. voids −1.75, urgency −1.88, nocturia episodes per day −0.52, and OBS NRS score −2.21 at 8 wks. Differences in mean changes between treatment and control groups for total no. voids −0.85, urgency −0.76, nocturia episodes per day −0.28, and OBS NRS score −1.16 at 8 wks.	235 (3 studies)	2 ⊕⊕⊕〇 (Moderate) 1⊕⊕〇〇 (Low)	Bladder-related QoL improved.
Sleep Disturbance		Mean change in sleep NRS 3.55 pts (min. 0.99, max. 6.1) at 12 wks. Survey 56% self-reported benefit in sleep quality.	816 (3 studies)	1 ⊕⊕⊕〇 (Moderate) 1 ⊕⊕〇〇 (Low)	MSQoL-54 improved by 25%.
